# Feasibility and preliminary efficacy of iConquerFear: a self-guided digital intervention for fear of cancer recurrence

**DOI:** 10.1007/s11764-022-01233-9

**Published:** 2022-07-25

**Authors:** Allan ‘Ben’ Smith, Adeola Bamgboje‐Ayodele, Sharuja Jegathees, Phyllis Butow, Britt Klein, Marj Salter, Jane Turner, Joanna Fardell, Belinda Thewes, Louise Sharpe, Lisa Beatty, Alison Pearce, Jane Beith, Daniel Costa, Orlando Rincones, Verena S. Wu, Frances L. Garden, Belinda E. Kiely, Karen Lim, Lisa Morstyn, Brigid Hanley, Rosemerry Hodgkin, Annette Beattie, Afaf Girgis

**Affiliations:** 1 Faculty of Medicine and Health, South West Sydney Clinical Campuses, University of New South Wales (UNSW Sydney), Liverpool, Australia; 2 Ingham Institute for Applied Medical Research, Liverpool Hospital, Locked Bag 7103, Liverpool, BC NSW 1871 Australia; 3 Biomedical Informatics and Digital Health, School of Medical Sciences, Faculty of Medicine and Health, The University of Sydney, Sydney, Australia; 4 Psycho‐Oncology Co‐operative Research Group (PoCoG), University of Sydney, Sydney, NSW Australia; 5 Health Innovation & Transformation Centre (HITC) & Biopsychosocial and eHealth Research & Innovation (BeRI), DVC‐R&I Portfolio, Federation University Australia, Churchill, Australia; 6 Department of Psychiatry, University of Queensland, Brisbane, Australia; 7 School of Clinical Medicine, Discipline of Paediatrics, UNSW Medicine & Health, UNSW Sydney, Sydney, Australia; 8 Western Sydney Youth Cancer Service, Westmead Hospital, Westmead, Australia; 9 School of Psychology, University of Sydney NSW, Sydney, Australia; 10 College of Education, Psychology & Social Work, Flinders University, Adelaide, Australia; 11 The Daffodil Centre, The University of Sydney, a joint venture with Cancer Council NSW, Sydney, Australia; 12 Sydney School of Public Health, University of Sydney, Sydney, Australia; 13 Chris O’Brien Lifehouse, Camperdown, NSW Australia; 14 South Western Sydney Local Health District, Campbelltown Hospital, Campbelltown, NSW Australia; 15 South Western Sydney Local Health District, Liverpool Hospital, Liverpool, NSW Australia; 16 Breast Cancer Network Australia (BCNA), Camberwell, Australia; 17 Cancer Council Queensland, Fortitude Valley, Australia; 18 Cancer Council Western Australia, Perth, Australia; 19 Cancer Council New South Wales, Sydney, Australia

**Keywords:** cancer, eHealth, fear of cancer recurrence, Oncology, online, psycho-oncology, self-management, survivorship, web-based

## Abstract

**Purpose:**

Approximately 50% of cancer survivors experience moderate-severe fear of cancer recurrence (FCR). Self-guided digital interventions have potential to address the high level of FCR-related unmet needs at scale, but existing digital interventions have demonstrated variable engagement and efficacy. This study aimed to evaluate the feasibility and preliminary efficacy of iConquerFear, a five-module self-guided digital FCR intervention.

**Methods:**

Eligible curatively treated breast cancer survivors were recruited. Participants reporting clinically significant FCR (≥ 13 on the Fear of Cancer Recurrence Inventory-Short Form; FCRI-SF) were given access to iConquerFear. Feasibility was indicated by > 50% of eligible participants enrolling in iConquerFear and recording moderate (≥ 120 min) or greater usage. Preliminary efficacy was evaluated via changes in self-reported FCR severity, anxiety, depression, intrusions and metacognitions from baseline to immediately and 3 months post-intervention.

**Results:**

Fifty-four (83%) of 65 eligible participants enrolled in iConquerFear; six subsequently withdrew. Thirty-nine (83%) participants recorded moderate (*n* = 24; 120–599 min) or high (*n* = 15; ≥ 600 min) usage. Engagement levels increased with participant age (*p* = 0.043), but were lower in participants with higher baseline FCR (*p* = 0.028). Qualitative feedback indicated engagement was sometimes limited by difficulties with navigation and relating to featured survivors. Participants reported significantly improved FCR (mean reduction (95%CI): baseline to post-intervention − 3.44 (− 5.18, − 1.71), baseline to 3-month follow-up − 4.52 (− 6.25, − 2.78), *p* =  < 0.001).

**Conclusion:**

iConquerFear is a feasible and potentially efficacious intervention for reducing FCR in breast cancer survivors. Easier navigation and more relatable examples may enhance engagement.

**Implications for Cancer Survivors:**

iConquerFear may help address moderate but burdensome FCR levels in cancer survivors.

## Introduction

There are an estimated 43 million people living with and beyond cancer (*hereafter referred to as cancer survivors*). Cancer survivors’ most commonly reported unmet supportive care need is for help managing fear of cancer recurrence (FCR) [1], defined as fear, worry or concern about cancer returning or progressing [2]. Approximately half of cancer survivors experience clinically significant FCR [3, 4], which is associated with psychological distress, poorer quality of life (QoL) and greater healthcare use [1, 5]. FCR is more prevalent amongst survivors who are female [1, 4, 6] and younger [1, 4, 6] and experience physical symptoms [1, 7–9]. If untreated, FCR can persist for many years, highlighting the need for intervention [1, 10, 11].

Existing interventions (mostly face-to-face) have demonstrated efficacy in reducing FCR and associated psychological distress and QoL impairment [12, 13]. For example, ConquerFear, a 5-session therapist-delivered treatment including contemporary cognitive behavioural therapy (CBT) approaches (i.e. Metacognitive and Acceptance and Commitment Therapy [14]), significantly improved FCR, cancer-related distress, anxiety and emotional QoL, with benefits maintained 6 months post-intervention [15]. However, barriers to face-to-face treatment, including distance, work, disease burden [16, 17] and more recently the COVID-19 pandemic limiting access to care, highlight the need for remotely delivered interventions [18]. Developing and evaluating more accessible FCR treatments have been identified as top international FCR research priorities [19]. Digital interventions can improve access to psychosocial support and facilitate self-management by survivors [17, 20, 21]. While survivors with severe FCR may need more intensive support [22], self-guided digital interventions may be appropriate for survivors with more moderate FCR levels [22, 23].

Digital interventions have demonstrated mixed results in reducing psychological burdens such as FCR [24]. A systematic review and meta-analysis of 20 articles found comparable efficacy of face-to-face and online interventions for improving the psycho-emotional state of cancer survivors [25]. A meta-analysis of FCR interventions found a larger effect size for face-to-face (*n* = 18; *g* = 0.38) versus remotely delivered (*n* = 3; *g* = 0.10) interventions, but the difference was not statistically significant [13], highlighting the need for further evaluation of remotely delivered FCR interventions. More recently, an online self-managed but clinician-supervised web-based CBT program (iCanADAPT) demonstrated efficacy in reducing FCR relative to treatment as usual (i.e. access to family doctor and/or local mental health services) [26]. However, trials of two other self-guided digital FCR interventions, FoRtitude [27] and CAREST [28], found comparable FCR reductions in the intervention and control groups, possibly due to their primary use of traditional CBT techniques generally found to be less efficacious in reducing FCR [13], or limited intervention engagement.

Digital intervention engagement, defined as both the subjective experience of flow involving interest and attention, and the extent of usage of a digitally based intervention [29], is a key indicator of digital intervention feasibility and likely efficacy [30]. In-depth engagement measures are rarely reported for digital health interventions [31], despite Consolidated Standards of Reporting Trials on eHealth (CONSORT-EHEALTH) recommendations [32]. Considering the variable non-usage and engagement levels reported [33, 34], it is critical to understand how participants engage with digital interventions and their various components and how engagement is related to psychological outcomes [35, 36].

To make FCR treatment more accessible, the efficacious face-to-face ConquerFear intervention [15] was adapted to a self-guided digital intervention (iConquerFear), and usability testing found that cancer survivors appreciated its flexibility and content design [22]. This study aimed to evaluate iConquerFear feasibility (*uptake and engagement levels*), and preliminary efficacy (*changes in FCR 10 and 22 weeks after gaining access*) with breast cancer survivors using the intervention as intended.

## Methods

### Trial design

This was a single-arm, non-blinded pilot trial approved by South-Western Sydney Local Health District (SWSLHD) Ethics Committee (reference number 2020/ETH0266).

### Participants

Women were eligible to participate if they had (i) completed hospital-based adjuvant treatment for breast cancer with curative intent, (ii) no evidence of recurrence, (iii) sufficient English proficiency for informed consent and intervention engagement, (iv) internet and email access and (v) scored ≥ 13 on the Fear of Cancer Recurrence Inventory-Short Form (FCRI-SF) [37] indicating moderate-severe FCR (measure described below) [38]. Exclusion criteria included moderately severe or severe depression, and/or suicidal ideation.

Participants were recruited from February to June 2021 using four methods: (1) referrals from Cancer Council New South Wales, Queensland or Western Australia information and support telephone services, (2) social media advertisements, (3) clinician referrals from SWSLHD Cancer Centres and (4) emails to the Breast Cancer Network Australia (BCNA) Review & Survey Group, an online database of breast cancer research volunteers.

### Sample size

Our target sample size was 60 women based on recommendations for pilot research [39–41]; hence, we aimed to recruit 75 women to allow for attrition.

### Procedure

Prospective participants were emailed a link to an online participant information sheet, consent form and FCRI-SF [37] and Patient Health Questionnaire (PHQ-9) screening questions [42] (further details in the outcomes section). A researcher contacted eligible participants to provide a study overview, answer questions and confirm participation. If consented, participants were given website access instructions and a unique login. The researcher called participants 1 week after recruitment to troubleshoot any technical issues experienced.

At their first login, participants were required to complete an online self-report baseline (T0) questionnaire. They were then recommended to sequentially complete iConquerFear modules over 10 weeks (i.e. one module per fortnight), although they had unrestricted access to all modules for 22 weeks. Participants were subsequently asked to complete post-intervention questionnaires 10 weeks (T1) and 22 weeks (T2) post-baseline.

### Intervention

iConquerFear was derived from the face-to-face ConquerFear intervention, which targets unhelpful beliefs about worry (i.e. metacognitions) that play a central role in the development and maintenance of severe FCR according to the cognitive processing model of FCR [43, 44]. It uses contemporary CBT techniques from acceptance and commitment therapy and metacognitive therapy to reduce unhelpful metacognitions and intrusiveness of thoughts about recurrence [45]. Psychoeducation and therapeutic strategies are delivered via didactic and interactive written and audio-visual material organised into 6 modules, an optional welcome module and five therapeutic modules: (1) goal setting, (2) attention training, (3) detached mindfulness, (4) learning to live well and manage worry, and (5) treatment summary and relapse plans [22]. To facilitate engagement, the strategies of tunnelling (sequential progression through activities) [46], personalised feedback, tailored automated email reminders, interactive reflections and quizzes were integrated into iConquerFear [22]. A detailed description of the intervention is published elsewhere [22].

### Measures

Self-reported *demographic and clinical characteristics* including age, gender, relationship status, cancer stage, time since diagnosis and treatment received were assessed at baseline.

### Primary outcomes

#### Uptake

Acceptable uptake was pragmatically defined as ≥ 50% of the breast cancer survivors reporting moderate or higher FCR in screening agreeing to participate in iConquerFear. This would equate to a large number participants in a subsequent trial or implementation of iConquerFear in view of the prevalence of FCR and lack of broadly accessible treatments.

#### Engagement

The primary engagement measure was total time spent using iConquerFear, as used in previous studies [33, 34]. We suggested that participants spend about an hour working through each module and expected that they would need to spend at least 120 min using iConquerFear (i.e. the approximate time needed to complete 2 therapeutic modules) to reduce FCR. Participants were categorised as low (0–119 min usage), moderate (120–599 min usage) and high (600 + min usage) users. Engagement was deemed acceptable if > 50% of participants were moderate or high users.

#### Preliminary efficacy

This was measured by pre- to post-intervention changes in FCR severity assessed using the FCRI-SF, a widely used and validated subscale of the FCRI [37], across all three time points T0, T1 and T2 [3, 47]. The response scale for each item is on a 5-point Likert scale, between 0 (‘*Not at all/Never*’) and 4 (‘*A great deal/Several times a day/hours/year*’)*.* Total scores range from 0 to 36, with higher scores indicating greater FCR [37, 48].

Reliable change in FCR was calculated following Jacobson and Truax’s recommendations using the published Cronbach alpha (*r* = 0.88) of the English version of the FCRI [49] and the standard deviation of baseline FCRI-SF scores. Post-intervention FCRI-SF score below the clinical cutoff of 13 indicated clinically significant change [37].

### Secondary outcomes

Secondary measures of *engagement* included *number of logins*, *page views*, and *module/intervention completion*. Dropout attrition (participants who withdrew from the study) and non-usage attrition (participants who did not complete any of Modules 1–5) were also measured [50]. Facilitators and barriers to iConquerFear engagement were captured through open-ended questions in the T1 survey and spontaneous participant feedback during follow-up.

*Anxiety* was measured using the 7-item *Generalised Anxiety Disorder-7 (GAD-7)* [51], which assesses the presence of generalised anxiety symptoms in the past 2 weeks using a 4-point Likert scale ranging from not at all (0) to nearly every day (3). Scores range from 0 to 21 and higher scores indicate worse anxiety.

*Intrusive thoughts* were measured using the 7-item intrusion subscale of the *Impact of Event Scale-Revised (IES-R)* [52, 53], which assesses distress caused by intrusive thoughts related to cancer in the past 7 days on a 5-point Likert scale ranging from not at all (0) to extremely (4). Scores range 0–28 and higher scores indicate more intrusive thoughts.

*Negative metacognitions* were measured using the 6-item negative beliefs about the worry subscale of the *Metacognitions Questionnaire (MCQ-30)* [54], which assesses agreement with statements of beliefs about worry on a 5-point Likert scale ranging from do not agree (0) to agree very much (4). Subscale scores range 6–24 and higher scores indicate more maladaptive metacognition.

*Depression* was screened for using the 9-item *Patient Health Questionnaire (PHQ-9)* [42], which assesses the frequency of depressive symptoms within the past 2 weeks using a 4-point Likert scale ranging from not at all (0) to nearly every day (3). Scores range from 0 to 27, with scores ≥ 15 indicating moderately severe/severe depression. Scores ≥ 1 on item 9 ‘Thoughts you would be better off dead or hurting yourself in some way’ indicated suicidal ideation.

### Data analysis

Quantitative analysis was conducted via IBM SPSS Statistics version 27. An independent *t*-test was used to compare mean FCRI-SF scores between those eligible and ineligible for the study. Descriptive statistics were used to summarise study sample characteristics. Differences in baseline characteristics between user groups (low, moderate, high) were assessed using chi-squared tests, independent *t*-tests, one-way ANOVA, Wilcoxon rank-sum or Kruskal–Wallis tests, depending on variable type. Linear mixed models were used to assess changes in FCRI-SF, GAD-7, IES-R and MCQ-30 scores from T0 to T1 and T2. The mixed models included a random intercept to account for repeated measurements, and participant age and years since diagnosis as potential confounders. The association between FCR change and intervention user group (low, moderate, high) and intervention completion (yes/no) was assessed by inclusion of these variables, respectively, into the mixed model. All tests were two-sided, and a *p* value of < 0.05 was deemed significant.

Qualitative feedback from open-ended questions and spontaneous participant comments during follow-up was thematically analysed [55] to determine barriers and enablers to iConquerFear engagement and identify potential improvements. Feedback was analysed using a six-step process: SJ reviewed the data, generated preliminary codes and grouped codes into overarching themes. ABS and AB then worked with SJ to review, finalise and write-up themes.

## Results

### Participants

**Fig. 1:**
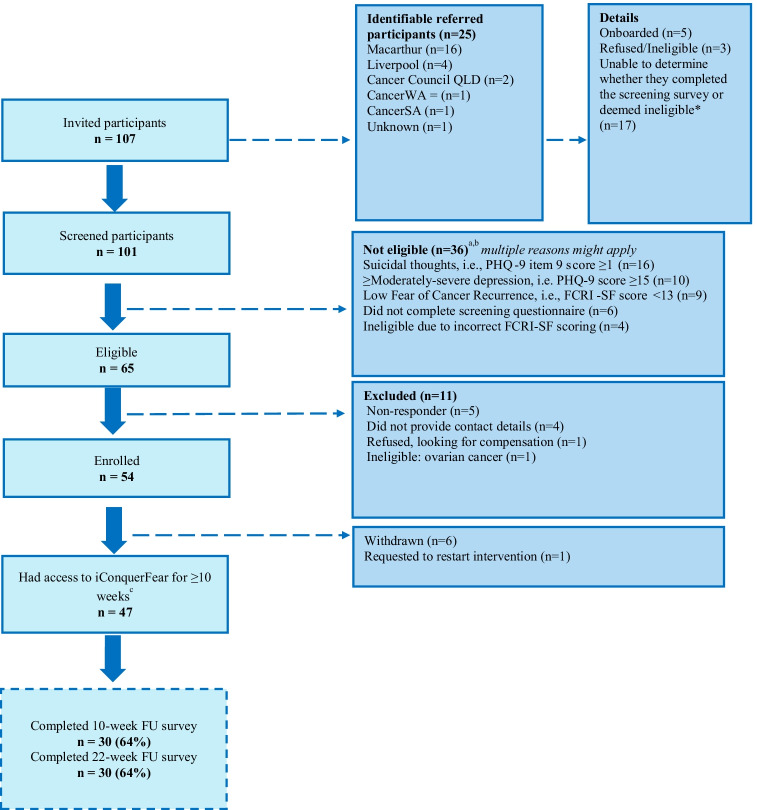
CONSORT participant recruitment flow diagram. *Unable to track which participants completed the screening questionnaire, as the survey does not request identifiable data. ^a^Multiple reasons may apply. ^b^One participant completed the screening questionnaire twice and was ineligible both times. ^c^Excluding all withdrawn participants. Forty-seven participants had a 10-week access to iConquerFear

From February to July 2021, 107 breast cancer survivors were invited to participate. Active recruitment ceased once our target of 75 screened participants was reached, but 101 ultimately completed screening, 83 (83%) reported FCRI-SF scores ≥ 13, and 65 (64.4%) were eligible, with 26 excluded due to moderate/severe depression, and/or suicidal ideation (see Fig. 1). There was no significant difference between FCRI-SF scores of eligible (mean = 21.08, standard deviation (SD) = 4.2) and ineligible participants (mean = 20.88, SD = 7.2, *p* = 0.901).

#### Feasibility (uptake)

Fifty-four (83.1%) of 65 eligible women agreed to participate in iConquerFear; thus, our feasibility target of enrolling at least 50% of eligible women was met. Six participants (11.3%) subsequently withdrew, three did not complete the baseline survey, and one was excluded as her data was lost when her account was reset to enable her to continue using the intervention after the study had ceased. Forty-four of 65 eligible participants (67.6%) completed the baseline survey and accessed the website. Thirty (63.8%) participants subsequently completed both the T1 (mean = 10.4, SD = 2.16 weeks post-T0) and T2 (mean = 25.3, SD = 1.5 weeks post-T0) questionnaire (see Fig. 1).

#### Sample characteristics

Eligible participants providing baseline data (*n* = 44) had a mean age of 55.3 years (SD = 9.8) and were a median of 4.0 years since diagnosis (IQR: 2.8.5). Most participants (70.4%) were married/partnered, had children (75.0%), had obtained a university degree (68.2%), were currently employed (68.2%), were born in Australia (68.2%) and primarily spoke English (86.4%). See Table 1 for other sample characteristics according to iConquerFear user group.

**Table 1:** Baseline demographics of all study participants and by user group

	Total sample^a^	User group			
Characteristic	*N* = 44	Low user (*n* = 5)	Moderate user (*n* = 24)	High user (*n* = 15)	*p* value^b^
Age, mean (SD)	55.3 (9.8)	50.8 (12.8)	53.3 (9.7)	60.1 (7.2)	0.043
Years since diagnosis, median (IQR)	4 (2, 8.5)	3 (2, 5)	4 (2, 8.5)	6 (3.5, 9)	0.313
FCR baseline, median (IQR)	20 (18, 23)	23.5 (20, 27.5)	20 (18.5, 23)	20 (17.5, 22)	0.028
Relationship					
Single	13 (29.6)	0 (0.0)	6 (25.0)	7 (46.7)	0.116
Married/Partnered	31 (70.4)	5 (100.0)	18 (75.0)	8 (53.3)	
Education					
University	30 (68.2)	3 (60.0)	17 (70.8)	10 (66.7)	0.904
Not university	14 (31.8)	2 (40.0)	7 (29.2)	5 (33.3)	
Employment					
Not working	14 (31.8)	2 (40.0)	8 (33.3)	4 (26.7)	0.814
Working	30 (68.2)	3 (60.0)	16 (66.7)	11 (73.3)	
Country of birth					
Australia	30 (68.2)	3 (60.0)	17 (70.8)	10 (66.7)	0.904
Other	14 (31.8)	2 (40.0)	7 (29.2)	5 (33.3)	
Language spoken					
English	38 (86.4)	4 (80.0)	20 (83.3)	14 (93.3)	0.561
Other	6 (13.6)	1 (20.0)	4 (16.7)	1 (6.7)	
Aboriginal or Torres Strait Islander					
No	44 (100)	5 (100.0)	24 (100.0)	15 (100.0)	N/A
Children					
No	11 (25.0)	3 (60.0)	4 (16.7)	4 (26.7)	0.154
Yes	33 (75.0)	2 (40.0)	20 (83.3)	11 (73.3)	
Stage					
Unspecified	8 (18.8)	0 (0.0)	4 (16.7)	4 (26.7)	0.217
1	13 (29.6)	2 (40.0)	10 (41.7)	1 (6.7)	
2	17 (38.6)	2 (40.0)	7 (29.2)	8 (53.3)	
3	6 (13.6)	1 (20.0)	3 (12.5)	2 (13.3)	
Treatment received^c^					
Surgery	44 (100)	5 (100.0)	24 (100.0)	15 (100.0)	N/A
Chemotherapy	25 (56.8)	3 (60.0)	12 (50.0)	10 (66.7)	0.633
Radiotherapy	32 (72.7)	4 (80.0)	17 (70.8)	11 (73.3)	0.999
Hormonal	29 (65.9)	4 (80.0)	16 (66.7)	9 (60.0)	0.819
Herceptin	2 (4.7)	0 (0.0)	0 (0.0)	2 (13.3)	0.210
Time since treatment completion					
Within last 6 m	14 (31.8)	2 (40.0)	7 (29.2)	5 (33.3)	0.946
Within last 2 years	15 (34.1)	2 (40.0)	9 (37.5)	4 (26.7)	
Over 2 years ago	15 (34.1)	1 (20.0)	8 (33.3)	6 (40.0)	
Other psychological treatment					
No	29 (67.4)	3 (60.0)	13 (56.5)	13 (86.7)	0.129
Yes	14 (32.6)	2 (40.0)	10 (43.5)	2 (13.3)	

*SD*, standard deviation; *IQR*, interquartile range. ^a^Total sample is those that were included in the study and who had baseline measurements. ^b^*p* value from chi-square test, *t*-test or Kruskal-Wallis test as appropriate. ^c^Participants could choose 1 or more options

#### Feasibility (engagement)

Eight participants (17.0%) were classed as low users (0–119 min usage), 24 (51.1%) as moderate users (120–599 min usage) and 15 (31.9%) as high users (≥ 600 min usage). Thus, our target of greater than 50% moderate to high users was met. High users demonstrated greater engagement across all measures and were significantly more likely to complete the intervention (73%; *p* < 0.001).

Thirty-nine (83.0%) of 47 participants given 10-week intervention access completed at least the first therapeutic module; the other eight (17%) completed no modules. Thirty-three (70.2%) participants completed at least two out of five therapeutic modules; 17 (36.2%) participants completed Module 5 and the intervention as whole. The average percentage of iConquerFear completed was 64.5% (SD = 35.4). Median number of page views was 52.5 (IQR = 19–77), with a total of 464 logins (median = 8.0, IQR = 3–12), and a median login duration of 73 min.

### Engagement correlates

#### User groups

As shown in Table 1, user group membership was significantly associated only with age (*p* = 0.043) and baseline FCR (*p* = 0.028). High users were older and had lower baseline FCR. Compared to moderate users:

1. Older age was associated with being a high user (OR = 1.09, 95% CI 1.005–1.186, *p* = 0.038);
2. Higher baseline FCR increased the likelihood of being a low user (OR = 1.26, 95% CI 1.004–1.585, *p* = 0.046).

### Preliminary efficacy

#### Primary outcome

For the 27 participants with FCRI-SF data at all three time points, linear mixed models analysis showed FCR severity significantly decreased over time from T0 (mean = 19.93, SD = 3.9) to T1 (mean = 16.48, SD = 5.2, mean reduction (95%CI): − 3.44 (− 5.18, − 1.71), *p* = 0.0002, Cohen’s *d* = 0.74) and further decreased from T0 to T2 (mean = 15.41, SD = 4.8, mean reduction (95%CI): − 4.52 (− 6.25, − 2.78), *p* =  < 0.001, Cohen’s *d* = 1.0) (Table 2). The significant reduction in FCR remained when the model was adjusted for age and years since diagnosis, respectively. Age and years since diagnosis were not associated with FCR (age beta: − 0.042 95%CI: (− 0.21, 0.13), *p* = 0.616; years since diagnosis beta: − 0.09 95%CI (− 0.44, 0.26), *p* = 0.598).

**Table 2:** Linear mixed model regression results for the association of FCR, anxiety, intrusive thoughts and negative metacognitions with time, age, years since diagnosis and usage characteristics (*n* = 27)

	FCR		Anxiety		Intrusive thoughts		Negative metacognitions	
	Beta (95%CI)	*p* value	Beta (95%CI)	*p* value	Beta (95%CI)	*p* value	Beta (95%CI)	*p* value
T0: Baseline	Ref							
T1: Post-intervention	− 3.44 (-5.18, − 1.71)	0.0002	− 1.07 (− 2.69, 0.52)	0.18	− 0.32 (− 0.54, − 0.11)	0.0039	− 1.78 (− 3.12, − 0.44)	0.0103
T2: 3 month follow-up	− 4.52 (− 6.25, − 2.78)	< 0.0001	− 1.63 (− 3.22, − 0.036)	0.045	− 0.26 (− 0.47, − 0.044)	0.019	− 1.89 (− 3.23, − 0.55)	0.0066
Current age	− 0.042 (− 0.21, 0.13)	0.616	− 0.055 (− 0.20, 0.092)	0.45	− 0.011 (− 0.03, 0.0082)	0.25	− 0.036 (− 0.17, 0.097)	0.58
Years since diagnosis	− 0.09 (− 0.44, 0.26)	0.598	− 0.035 (− 0.34, 0.27)	0.81	− 0.011 (− 0.051, 0.029)	0.58	0.018 (− 0.26, 0.29)	0.90
Intervention user group								
Moderate	Ref							
High	− 2.66 (− 5.66, 0.34)	0.08	− 1.42 (− 4.13, 1.30)	0.29	− 0.23 (− 0.58, 0.12)	0.19	− 1.32 (− 3.76, 1.13)	0.28
Intervention completion								
No	Ref							
Yes	− 3.83 (− 6.54, − 1.11)	0.008	− 1.45 (− 4.11, 1.22)	0.27	− 0.26 (− 0.61, 0.08)	0.13	0.28 (− 2.19, 2.74)	0.82

All models included FCR scores as the outcome and time as a fixed effect. The beta coefficients for the other variables are from separate models that include the variable, FCR scores and time

#### Reliable and clinically significant change in FCR

**Fig. 2:**
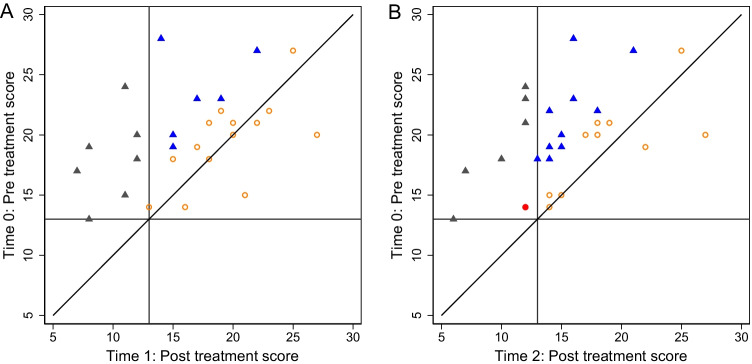
Reliable and clinically significant change in fear of cancer recurrence (FCRI-SF scores). Figure description: change in fear of cancer recurrence between (**A**) pre- and post-treatment (T0 to T1) and (**B**) pre-treatment and 3-month follow-up (T0 to T2), for participants (*n* = 27) who completed the FCRI-SF at T0, T1 and T2. The diagonal line indicates no change. The horizontal and vertical lines indicate the FCRI-SF clinical cutoff of ≥ 13 pre- and post-treatment. Orange circle: no reliable or clinically significant change. Blue triangle: reliable, but not clinically significant change. Grey triangle: reliable and clinically significant change. Red circle: not reliable, but clinically significant change

The reliable change criterion was calculated to be 3.68 (Cronbach alpha = 0.88 from literature, and baseline survey standard deviation = 3.82). Figure 2 shows the change in FCR from baseline (T0) to (A) post-intervention (T1) and (B) 3-month follow-up (T2), for the 27 participants with FCRI-SF data at all three time points. Seven (25.9%) participants experienced reliable and clinically significant improvement at T1 and six (22.2%) at T2.

#### Factors associated with level of FCR change

There was no difference in FCR change according to intervention user group (high vs moderate, *p* = 0.08), but those who completed the intervention had significantly lower FCR than those who did not (mean difference: − 3.8, 95%CI: − 6.5, − 1.1, *p* = 0.008).

#### Secondary outcomes

Compared to baseline (T0), there were significant decreases in *intrusive thoughts* at T1 (mean difference: − 0.32, 95%CI: − 0.54, − 0.11, *p* = 0.004) and T2 (mean difference: − 0.25, 95%CI: − 0.47, − 0.04, *p* = 0.02); *negative metacognitions* at T1 (mean difference: − 1.78, 95%CI: − 3.12, − 0.44, *p* = 0.01) and T2 (mean difference: − 1.89, 95%CI: − 3.23, − 0.55, *p* = 0.007); and *anxiety* at T2 (mean difference: − 1.63, 95%CI: − 3.22, − 0.04, *p* = 0.045), but not T1 (mean difference: − 1.0741, 95%CI: − 2.67, 0.52, *p* = 0.18). Significant reductions in secondary outcomes remained when the model was adjusted for age and years since diagnosis, respectively. See Table 2 for further details.

### Qualitative evaluation of iConquerFear

Thirty-two of 47 participants gave qualitative feedback via open-ended questions and spontaneous comments during follow-up. Three key themes were generated from thematic analysis: acceptability of iConquerFear, challenges with engagement and recommendations for improvement. Participants largely perceived iConquerFear to be an informative, reassuring, useful and effective tool for managing FCR. However, engagement was sometimes limited by technical and time barriers, and some wanted greater signposting of resources and demographic diversity of cancer survivors featured to make content easier to navigate and relate to. See Table 3 for description of each theme and illustrative quotes.

**Table 3:** Qualitative evaluation of iConquerFear

Themes, description and illustrative quotes
Acceptability of iConquerFear Participants reported overall satisfaction with iConquerFear and perceived it to be an informative, reassuring, useful and effective tool for managing FCR: *I liked it a lot it certainly helped me to face some of my worries or concerns that I am now able to let go* (WA005) *I am starting to sleep a little better & not stress out as much or worry at night about my fear of cancer returning* (NSW002) By empowering women to self-manage their FCR, iConquerFear was reported to help women transition back into the routine of daily life, regardless of their stage of survivorship: *I learned ways to become more aware and how to manage my thoughts and fears about cancer* (NSW007) Participants also expressed satisfaction in the ability to revisit the intervention to download resources for continued use: *l have downloaded the resources so l can use them, in particular so l can continue doing the attention training* (WA007)
Challenges with engagement Reported engagement barriers included technical issues, access difficulties and some content not resonating with personal experience Several participants noted system glitches, such as inability to hear sound recordings and input responses to questions. Some found email prompts to engage with iConquerFear overly frequent. Participants also reported the intervention was not particularly mobile friendly: *It was trickier to use on a mobile phone than on a laptop. The scrolling button on the very right-hand side is difficult to see, but that may be just my computer* (NSW001) *It will be good in the future when the clunkiness of the system can be removed so the user interaction is more seamless* (NSW003) Multiple participants identified limited time as an engagement barrier, and one participant reported limited motivation to complete recommended tasks: *I wasn’t able to keep up with each module in the required timeframe due to work and home pressures, and it was annoying not being able to access modules I had missed* (ACT002) The potential for iConquerFear to trigger distress, was also raised as a concern: *I found the initial module triggered my fears of recurrence and I felt upset more generally. This seemed to lead to a significant period of stress and fatigue for me* (WA002) Some participants reported certain content did not resonate with them and had particular difficulty relating to the breast cancer survivor featured in videos throughout iConquerFear: *The words from the cancer survivor—everyone*’*s experience is so different, and she is such a different age bracket to me that I found it hard to relate to anything she was saying—I appreciate what the section is trying to do but it*’*s very hard to put a person in that role who will resonate with all people, and she really didn*’*t with me unfortunately* (NSW008)
Recommendations for improvement A few participants expressed a need for more resources on lifestyle and family support. In terms of content design, participants recommended making resources easier to find to aid navigation, and to have a wider representation of cancer survivors to allow for more relatable content: *Perhaps drawing from a range of age groups in the images used—young women, middle aged women, older women. That would have made it more relatable for me* (SA001)

## Discussion

There is a critical need for scalable FCR interventions to address cancer survivors’ great unmet need for managing FCR. This pilot study evaluated the feasibility and preliminary efficacy of iConquerFear, a self-guided digital intervention, to reduce FCR. Feasibility was demonstrated by > 80% of eligible participants enrolling in iConquerFear and > 80% recording moderate to high use. Older participants were more likely to engage with iConquerFear, while those with higher baseline FCR were less likely. On average, participants reported significant reductions in FCR from baseline to post-intervention of a medium to large effect size, with improvements maintained 3 months post-intervention; about one-quarter reported clinically significant improvements at both post-intervention time points. Change in FCR was not significantly associated with intervention engagement, perhaps due to limited statistical power. Suggested changes to increase iConquerFear engagement and efficacy included easier navigation and greater demographic diversity of featured cancer survivors.

The potential of online FCR self-management interventions is unlikely to be realised unless they successfully engage cancer survivors. iConquerFear engagement was greater than similar interventions like CAREST, where limited usage was suggested to explain its lack of efficacy [28], and comparable to FoRtitude, which also averaged 8 logins and about 60% of participants completing two-thirds of the intervention [27]. The relatively high iConquerFear engagement levels may be due to its theory- and person-based development, which facilitated flexible access, easy navigation and content satisfaction/engagement using techniques such as content tunnelling, interactive exercises and tailored feedback [22].

While initial iConquerFear engagement was relatively high, as with other digital interventions (e.g. FoRtitude [27]), it declined, with only 36% of participants completing all modules, compared with 67% of face-to-face ConquerFear participants who completed all five sessions [15]. We predicted that participants would need to spend at least 2 h using iConquerFear (i.e. the estimated time to complete 2 modules) to derive benefit, based on the ConquerFear RCT and iConquerFear usability study feedback, but there is limited evidence to suggest what ‘dose’ of digital self-guided interventions is needed to have an effect [56]. Some iConquerFear participants may have benefitted from early intervention components, such as the values clarification card sort task in Module 1, and chosen to focus on applying that learning, rather than continue with iConquerFear. The FoRtitude RCT found higher intervention use was related to greater FCR reduction [27]. Our study did not find this association, perhaps because it was not powered to detect it, or it may reflect the variable benefit participants gained from different intervention components and amounts.

### Correlates of engagement

Older age was associated with greater iConquerFear engagement, which was unexpected as younger age is associated with greater acceptability of online self-management amongst cancer survivors [57]. However, age has demonstrated equivocal associations with engagement in online psychological interventions generally [58]. Many studies reporting correlations between older age and lower engagement were conducted several years ago. These studies may not reflect increasing digital literacy in older people in the past decade, with older cancer survivors more often seeking health guidance on the Internet [59], enabling them to better engage with digital interventions. Online recruitment (e.g. via email listserv) of some participants and the generally high education levels of our sample may also have produced a more digitally literate sample of cancer survivors, with older women having greater opportunities to engage with iConquerFear. Qualitative feedback indicated that COVID-19 negatively affected engagement by younger women, who reported greater home pressures and limited time consistent with the greater burden of caregiving and home duties during lockdowns on younger women generally [60].

Higher baseline FCR was also associated with lower iConquerFear engagement. This is consistent with a study of uptake and adherence to online mindfulness-based cognitive therapy (eMBCT), which found non-users had higher baseline FCR than users [33]. In general, mixed associations between baseline symptom severity and engagement with online psychological interventions have been found [58]. Higher FCR has been associated with greater avoidance coping [61], which may lead to poor intervention engagement. Participants with higher FCR may also have perceived iConquerFear as insufficiently intensive to address their fears, limiting engagement. Qualitative feedback indicated some with higher FCR found the self-guided aspect of iConquerFear challenging, as they had to confront their FCR with limited perceived support. Clearer explanation of what iConquerFear involves, how to best use the intervention and more prominent links to in-person support may help increase uptake and engagement. Self-guided interventions may be best suited to people with moderate FCR, with more intensive approaches better suited to those with severe FCR [62]. Stepped/matched care FCR treatment models, where patients are allocated to interventions they are most likely to engage with and benefit from based on FCR severity, have demonstrated promise in FCR management [63].

Another option to augment engagement with interventions like iConquerFear and increase their suitability for those with more severe FCR, without overly limiting their scalability, could be to add therapist guidance. In a pilot RCT of iCanADAPT, an online self-guided, but therapist-supervised CBT program, participants averaged 64.3 min (SD = 40 min) of therapist contact; 40/52 (77%) completed all eight treatment lessons and experienced significantly greater reductions in total FCR than treatment as usual controls immediately post-intervention and at 3 months follow-up [26]. Telecoaching (i.e. 4 weekly telephone-based motivational interviews) promoted greater engagement with FoRtitude, which was associated with larger FCR reductions [27]. Relatively minimal contact with a coach or therapist may improve engagement (and efficacy in turn) and a RCT of therapist-guided iConquerFear is underway in Denmark [64].

### Preliminary efficacy

iConquerFear demonstrated preliminary efficacy, with medium-large-sized FCR reductions at post-intervention (*d* = 0.74) and 3 months follow-up (*d* = 1.0), similar to within-group reductions for face-to-face ConquerFear participants (*d* = 0.77) [15]. As in the ConquerFear RCT, there were also significant reductions in anxiety, which, like FCR improvements, were maintained at 3-month follow-up. Comparison with other online FCR intervention trials is complicated by the different measures, methods and analyses used. The preliminary efficacy of iConquerFear may be due to its contemporary CBT approach, shown to be more effective than traditional CBT techniques [13], such as those largely used in CAREST [28] and FoRtitude [27]. Reductions in FCR were accompanied by decreases in maladaptive metacognitions and distress related to intrusive thoughts about cancer, previously shown to partially mediate the effects of ConquerFear on FCR [45]. Our pilot study was not powered to test mediation models and with no control group decreased maladaptive metacognitions and intrusive thoughts cannot be causally attributed to the intervention. However, concurrent reductions in FCR, unhelpful metacognition and intrusive thoughts suggest a possible mechanism for the observed improvements in FCR consistent with the cognitive processing model of FCR [43].

Despite promising FCR reductions on average, only a quarter of participants reported clinically significant improvement. This could partly be due to the criteria for clinically significant change used, which meant that some participants reporting severe FCR initially experienced sizeable reductions in FCR without going below the clinical cutoff of 13. Establishing a minimally important difference on the FCRI-SF would mitigate this problem. Issues with longer term survivors (approximately 5 years post-diagnosis in our sample) being more likely to score highly on certain FCRI-SF items (e.g. ‘How long have you been thinking about the possibility of cancer recurrence?’) may also increase the difficulty of reducing scores below the clinical cutoff [3]. FCR scores may also have been inflated for many participants recruited shortly before a major COVID outbreak in Australia, as concerns about the impact of COVID on cancer management have been associated with FCR [65].

### Potential improvements

iConquerFear was generally seen as an engaging and useful tool for self-managing FCR, as per previous usability testing, but improvements were suggested. Participants’ main critique was that some content was ‘impersonal’ or ‘unrelatable’, a common challenge for self-guided interventions. We tried to personalise iConquerFear by giving participants feedback tailored according to information provided while using iConquerFear, but much of the intervention content was fixed and due to budget constraints, a single breast cancer survivor featured in videos. In future modifications, the content, including the demographics and experiences of cancer survivors featured, should ideally be tailored according to user characteristics, as tailoring improves engagement [66].

### Strengths and limitations

iConquerFear was adapted from the efficacious face-to-face ConquerFear intervention using a person-based approach, maximising the likelihood of feasibility and preliminary efficacy being shown in this pilot study. We screened and evaluated participants’ FCR using a well-validated measure and used comprehensive measures of iConquerFear engagement. A key study limitation was the lack of a control group, meaning that observed reductions in FCR cannot be attributed to iConquerFear specifically and may be due to expectation effects or spontaneous recovery. The relatively small sample also limited statistical power to identify factors associated with the preliminary efficacy of iConquerFear, such as intervention usage, and control for potential confounders. Future research could use a Multiphase Optimisation Strategy (MOST) framework, as per the FoRtitude trial [27], to efficiently evaluate the efficacy of both the intervention overall and individual intervention components. This may aid development of a shorter version of iConquerFear including only the most effective components, which may augment intervention engagement and benefit.

iConquerFear was evaluated in a well-educated sample of survivors of localised breast cancer who were largely Australian-born and spoke English as their first language, so our study results may not generalise to other cancer survivors. While our finding that older age was associated with greater iConquerFear engagement challenges the notion that digital health interventions may not be suitable for older cancer survivors, the use of online recruitment may have resulted in a more digitally literate sample of cancer survivors, despite recruiting participants from multiple sources to try and increase diversity. There is a critical need to evaluate digital health interventions in a range of participants, including from culturally and linguistically diverse backgrounds, with rare and advanced cancers, and varied degrees of digital literacy to ensure that they deliver on their promise to increase the accessibility and equity of cancer survivorship care when they are implemented in routine care.

## Conclusion

iConquerFear is a feasible self-guided digital intervention for reducing FCR, as evidenced by high engagement levels. Engagement could be further improved through easier navigation and increased tailoring of content to match user characteristics. Preliminary efficacy in reducing FCR out to 3 months needs RCT confirmation, ideally with participants from diverse backgrounds. iConquerFear may be most suitable for people with moderate FCR. Future integration into FCR stepped care models including more intensive options should be considered.

## Data Availability

Data available within the data or its supplementary materials.
